# Transfer of Learning in the Convolutional Neural Networks on Classifying Geometric Shapes Based on Local or Global Invariants

**DOI:** 10.3389/fncom.2021.637144

**Published:** 2021-02-19

**Authors:** Yufeng Zheng, Jun Huang, Tianwen Chen, Yang Ou, Wu Zhou

**Affiliations:** ^1^Department of Data Science, University of Mississippi Medical Centre, Jackson, MS, United States; ^2^Department of Otolaryngology-Head and Neck Surgery, University of Mississippi Medical Centre, Jackson, MS, United States

**Keywords:** ShapeNet, topological perception, convolutional neural network (CNN), global feature, shape classification

## Abstract

The convolutional neural networks (CNNs) are a powerful tool of image classification that has been widely adopted in applications of automated scene segmentation and identification. However, the mechanisms underlying CNN image classification remain to be elucidated. In this study, we developed a new approach to address this issue by investigating transfer of learning in representative CNNs (AlexNet, VGG, ResNet-101, and Inception-ResNet-v2) on classifying geometric shapes based on local/global features or invariants. While the local features are based on simple components, such as orientation of line segment or whether two lines are parallel, the global features are based on the whole object such as whether an object has a hole or whether an object is inside of another object. Six experiments were conducted to test two hypotheses on CNN shape classification. The first hypothesis is that transfer of learning based on local features is higher than transfer of learning based on global features. The second hypothesis is that the CNNs with more layers and advanced architectures have higher transfer of learning based global features. The first two experiments examined how the CNNs transferred learning of discriminating local features (square, rectangle, trapezoid, and parallelogram). The other four experiments examined how the CNNs transferred learning of discriminating global features (presence of a hole, connectivity, and inside/outside relationship). While the CNNs exhibited robust learning on classifying shapes, transfer of learning varied from task to task, and model to model. The results rejected both hypotheses. First, some CNNs exhibited lower transfer of learning based on local features than that based on global features. Second the advanced CNNs exhibited lower transfer of learning on global features than that of the earlier models. Among the tested geometric features, we found that learning of discriminating inside/outside relationship was the most difficult to be transferred, indicating an effective benchmark to develop future CNNs. In contrast to the “ImageNet” approach that employs natural images to train and analyze the CNNs, the results show proof of concept for the “ShapeNet” approach that employs well-defined geometric shapes to elucidate the strengths and limitations of the computation in CNN image classification. This “ShapeNet” approach will also provide insights into understanding visual information processing the primate visual systems.

## Introduction

Over the past six decades, investigations of visual system anatomy, physiology, psychophysics and computation have resulted in a general model of vision, which begins from extracting the local features of the retinal images in the lower visual areas [e.g., Lateral Geniculate Nucleus (LGN), V1], then integrates the local features to extract the *global features* in the higher visual areas (e.g., V4 and IT) (Hubel and Wiesel, 1977; Marr, 1982). The convolutional neural networks (CNNs) are primarily inspired by this local-to-global hierarchical architecture of the visual pathways. Similar visual neurons that encode visual properties of a special region of the visual field (i.e., receptive fields), the CNN units perform computations using inputs from special regions of the image and the receptive fields of units at different CNN layers exhibit different properties. With roots in biology, math and computer science, the CNNs have been the most influential innovation in the field of computer vision and artificial intelligence (AI). The CNNs can be trained to classify natural images with accuracies comparable to or better than humans. It has become the core of top companies' services, such as Facebook's automatic tagging algorithms, Google's photo search, and Amazon's product recommendations.

Despite the commercial success of the CNNs, however, little is known about how the CNNs achieve image classification and whether there are inherent limitations. This knowledge is important for avoiding catastrophic errors of the CNN applications in critical areas. To provide insight into the CNNs limitations vs. advantages, we developed a new approach of training and testing the CNNs, which is an alternative to the popular ImageNet approach. The body of literatures (Liu et al., 2018; Hussain et al., 2018) reported the performance of CNNs transfer learning based on image classification. Instead of using natural images to train and test the CNNs, we employed geometric shapes as the training and testing datasets (Zheng et al., 2019). In addition to training the CNNs to perform shape classification tasks, we focused on assessing how the CNN learning in the training datasets is transferred to new datasets (i.e., transfer datasets), which have new shapes that share local/global features with the training datasets. By varying the train and transfer datasets, we will be able to determine whether a local/global feature is extracted by the CNNs during the learning process. The goal was to directly test two hypotheses on CNN image classification. The first hypothesis is that transfer of learning based on local features is higher than transfer of learning based on global features. The second hypothesis is that the CNNs with advanced architectures have higher transfer of learning based on global features. In this study, we analyzed transfer of learning in four representative CNN models, i.e., AlexNet, VGG-19, ResNet-101, and InceptionResNet-v2, which have been trained on the ImageNet and achieved high accuracies in classifying natural images. We found that the results rejected the two hypotheses. Although preliminary, the present study provided proof of concept for this new “ShapeNet” approach.

## Convolutional Neural Networks

### Overview of Convolutional Neural Networks

In this study, four representative CNN models were tested, including the first deep-CNN (AlexNet), a significantly improved CNN model (VGG-19), and two milestones of the advanced CNNs (ResNet-101 and Inception-ResNet-v2). Their characteristics are summarized in Table 1. All the CNN models take color images as inputs, thus three-channel grayscale images are created for training. All shape images are scaled to proper size according to each model prior to training and testing. The four CNNs have been pretrained with the ImageNet.

**Table 1:** Summary of the four CNN models.

**CNN Model**	**AlexNet**	**VGG-19**	**ResNet-101**	**Inception-ResNet-v2**
Top-1 Accuracy (on ImageNet)	57.1%	71.3%	77.1%	80.0%
Number of Layers	8	19	101	164
Number of Parameters	63M	143.7M	44.5M	56M
Input Image Size	227 × 227 × 3	224 × 224 × 3	224 × 224 × 3	299 × 299 × 3

### AlexNet

AlexNet (Krizhevsky et al., 2012) is a deep CNN for image classification that won the ILSVRC (The ImageNet Large Scale Visual Recognition Challenge) 2012 competition (Russakovsky et al., 2015). It was the first model performed so well on the historically difficult ImageNet. AlexNet has eight layers with a total of 63 M parameters (Table 1). The first five layers are convolutional and the last three layers are fully connected. The AlexNet uses *Relu* instead of *Tanh* to add non-linearity and accelerates the speed by six times at the same accuracy. It uses *dropout* instead of *regularization* to deal with overfitting. AlexNet was trained using batch *stochastic gradient descent* (SGD), with specific values for *momentum* and weight decay.

### VGG-19

Simonyan and Zisserman (2014) created a 19-layer (16 conv., 3 fully-connected) CNN that strictly used 3 × 3 filters with stride and pad of 1, along with 2 × 2 max-pooling layers with stride 2, called VGG-19 model^1^ To reduce the number of parameters in such a deep network, it uses small 3 × 3 filters in all convolutional layers and best utilized with its 7.3% error rate. The VGG-19 has a total of 143.7M parameters. As the winner of ILSVRC 2015, it is one of the most influential models because it reinforced the notion that the CNNs need to have a deep network of layers for hierarchical representation of visual data.

### ResNet-101 and Inception-ResNet-v2

As the winner of ILSVRC 2015, the ResNet-101 (He et al., 2016) has 101 layers, consisting 33 three-layer *residual* blocks plus input and output layers. *Identity connections* learn incremental, or residual, representations, which creates a path for back-propagation. The identity layers gradually transform from simple to complex. Such evolution occurs if the parameters for the *f* (*x*) part begin at or near zero. The residual block helps overcome the hard training problem in DeepNet (> 30 layers) due to vanishing gradients. The ResNet-101 model uses 3 × 3 filters with stride of 2, and 3 × 3 max-pooling layers with stride 2.

Inception-ResNet-v2 is a hybrid inception version with residual connections, which leads to dramatically improved recognition performance and training speed in contrast with the inception architecture (Szegedy et al., 2017). The inception model uses variant kernel size (in v1) to capture the features from variant object size and location, introduces batch (weight) normalization (in v2) and factorizing convolutions (in v3), and uses bottleneck layers (1 × 1) to avoid a parameter explosion. The combination of the two most recent ideas: residual connections (Szegedy et al., 2016) and the latest revised version of the inception architecture (Szegedy et al., 2016). It is argued that residual connections are inherently important for training very deep architectures (He et al., 2016). Since inception networks tend to be very deep, it is natural to replace the filter concatenation stage of the inception architecture with residual connections. This would allow Inception to reap the benefits of the residual approach while retaining its computational efficiency.

## Experimental Results

### Experimental Design and Datasets

As shown in Table 2, there were 24 categories of shapes (540 images per category), which were generated in MatLab with variations created with transforms including translation, rotation and scaling. For the learning tasks, 85% of the learning datasets were used for training and 15% of the learning datasets were used for measuring validation accuracies, which are reported as learning accuracies. For the transfer tasks, classification accuracies in the transfer datasets are reported as transfer accuracies. Notice that the training datasets and the transfer datasets were different and separated. For example, in Experiment A.1, the CNNs were trained with squares and trapezoids, but never with rectangles. The four CNNs, which were pretrained with the ImageNet, were retrained with the learning datasets for 20 epochs.

**Table 2:** Examples of shapes for the learning and transfer tasks.

**Exp. #**	**Sample images for learning tasks**	**Sample images for transfer tasks**	**Description**
**A.1**	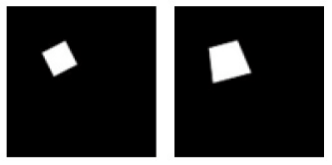	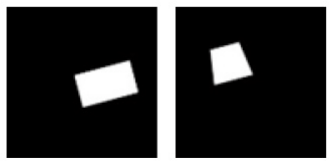	Learning: Square vs. Trapezoid Transfer: Rectangle vs. Trapezoid
**A.2**		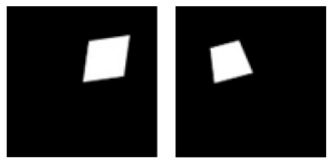	Learning: Parallelogram vs. Trapezoid Transfer: Rectangle vs. Parallelogram
**A.3**		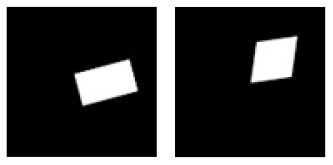	Learning: Square vs. Trapezoid Transfer: Rectangle vs. Trapezoid
**B.1**	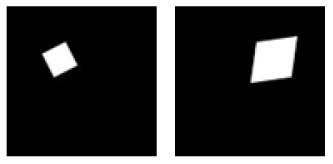	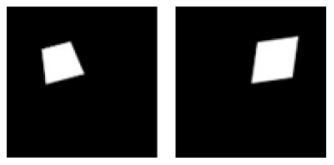	Learning: Square vs. Parallelogram Transfer: Trapezoid vs. Parallelogram
**B.2**		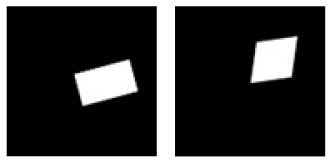	Learning: Square vs. Parallelogram Transfer: Rectangle vs. Parallelogram
**B.3**		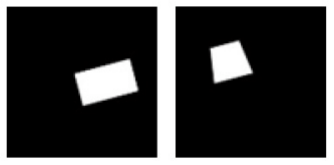	Learning: Square vs. Parallelogram Transfer: Rectangle vs. Trapezoid
**C.1**	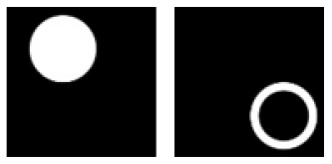	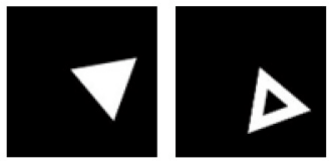	Learning: Disk vs. Ring Transfer: Triangle vs. Triangle-ring
**C.2**		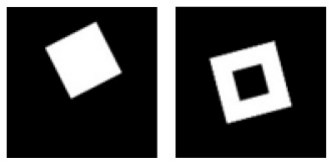	Learning: Disk vs. Ring Transfer: Square vs. Square-ring
**D.1**	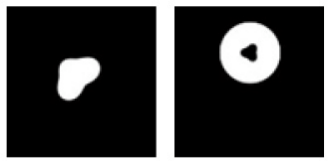	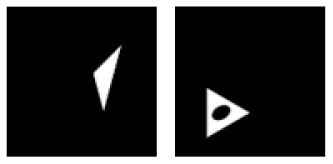	Learning: Irregular-disk vs. Irregular-ring Transfer: Irregular-triangle vs. Irregular-triangle-ring
**D.2**		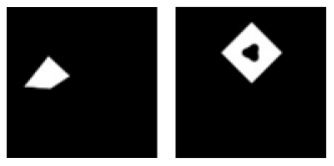	Learning: Irregular-disk vs. Irregular-ring Transfer: Irregular-square vs. Irregular-square-ring
**E**	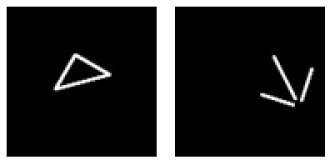	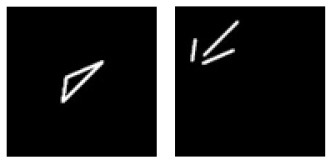	Learning: Isosceles-triangle vs. Disassembled-Isosceles-triangle Transfer: Irregular-triangle vs. Disassembled-irregular-triangle
**F**	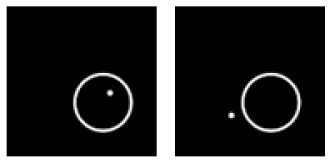	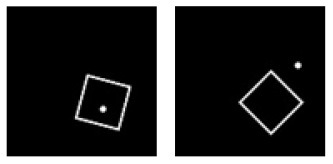	Learning: Dot-inside-circle vs. Dot-outside-circle Transfer: Dot-inside-square vs. Dot-outside-square
		

**Figure 1:**
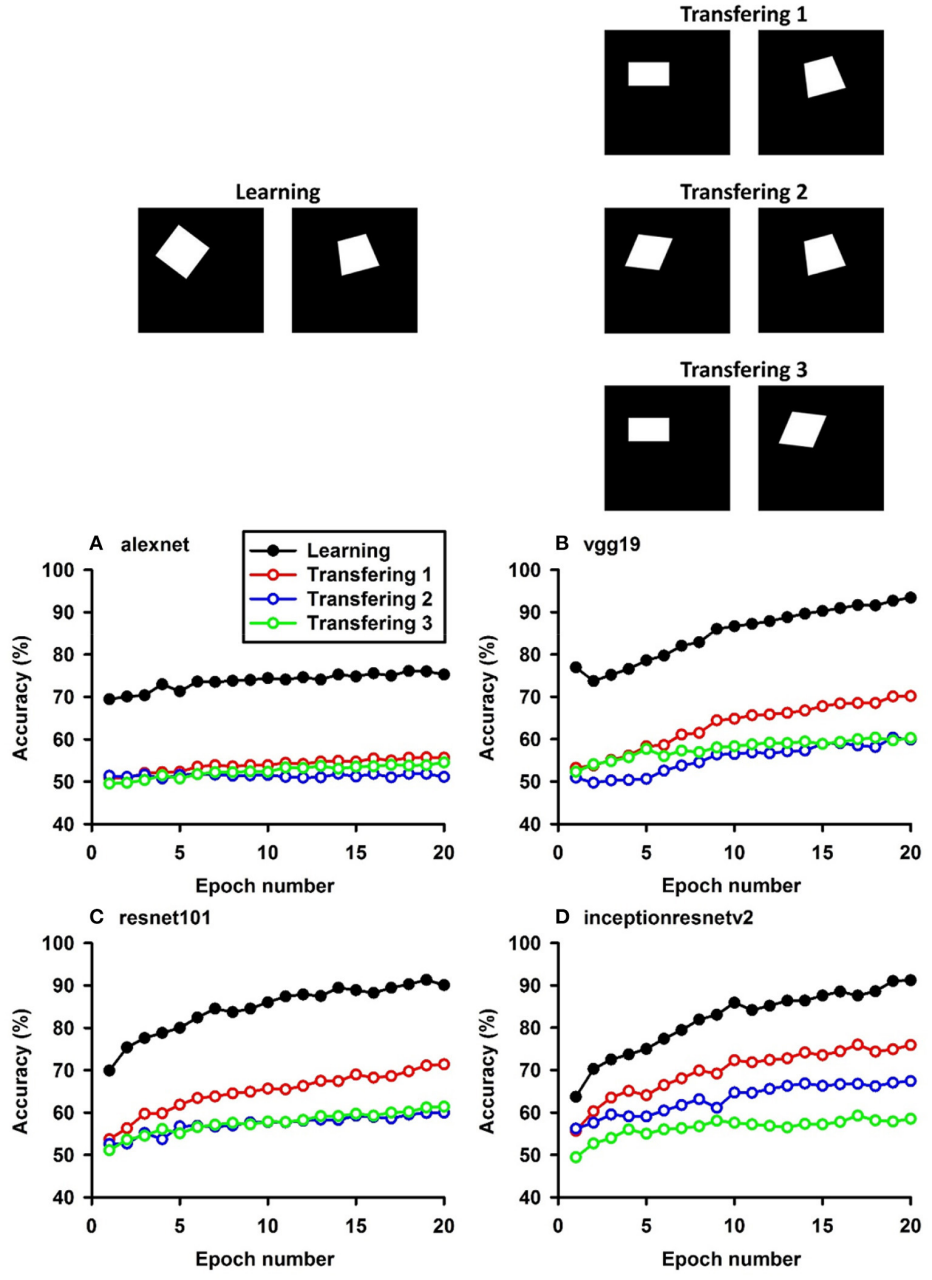
Experiment A. The top panels show the images used in learning and transfer tasks. The lower panel is learning and transfer accuracies as a function of training epochs for the four CNN models.

**Figure 2:**
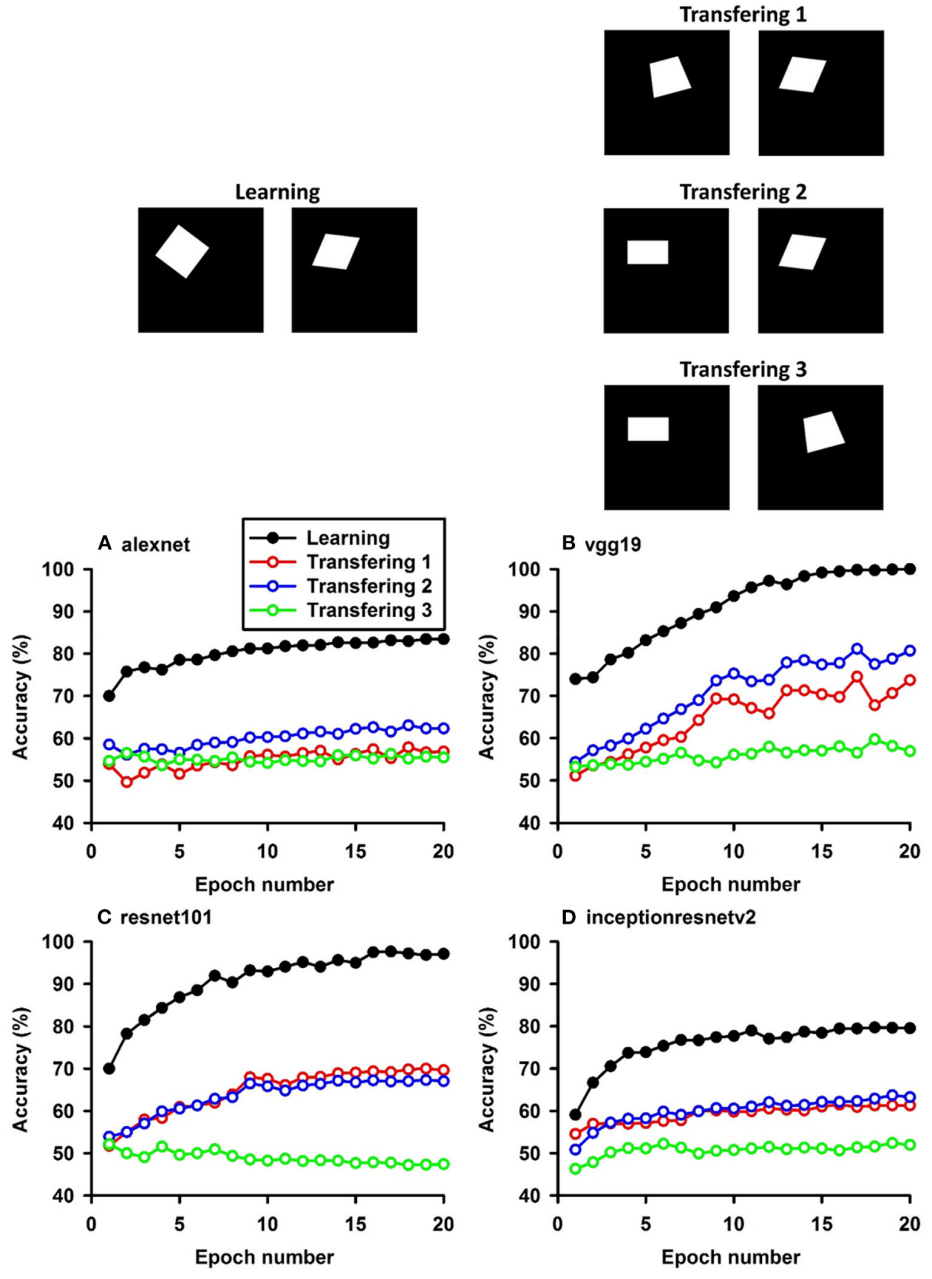
Experiment B. The top panels show the images used in learning and transfer tasks. The lower panel is learning and transfer accuracies as a function of training epochs for the four CNN models.

**Figure 3:**
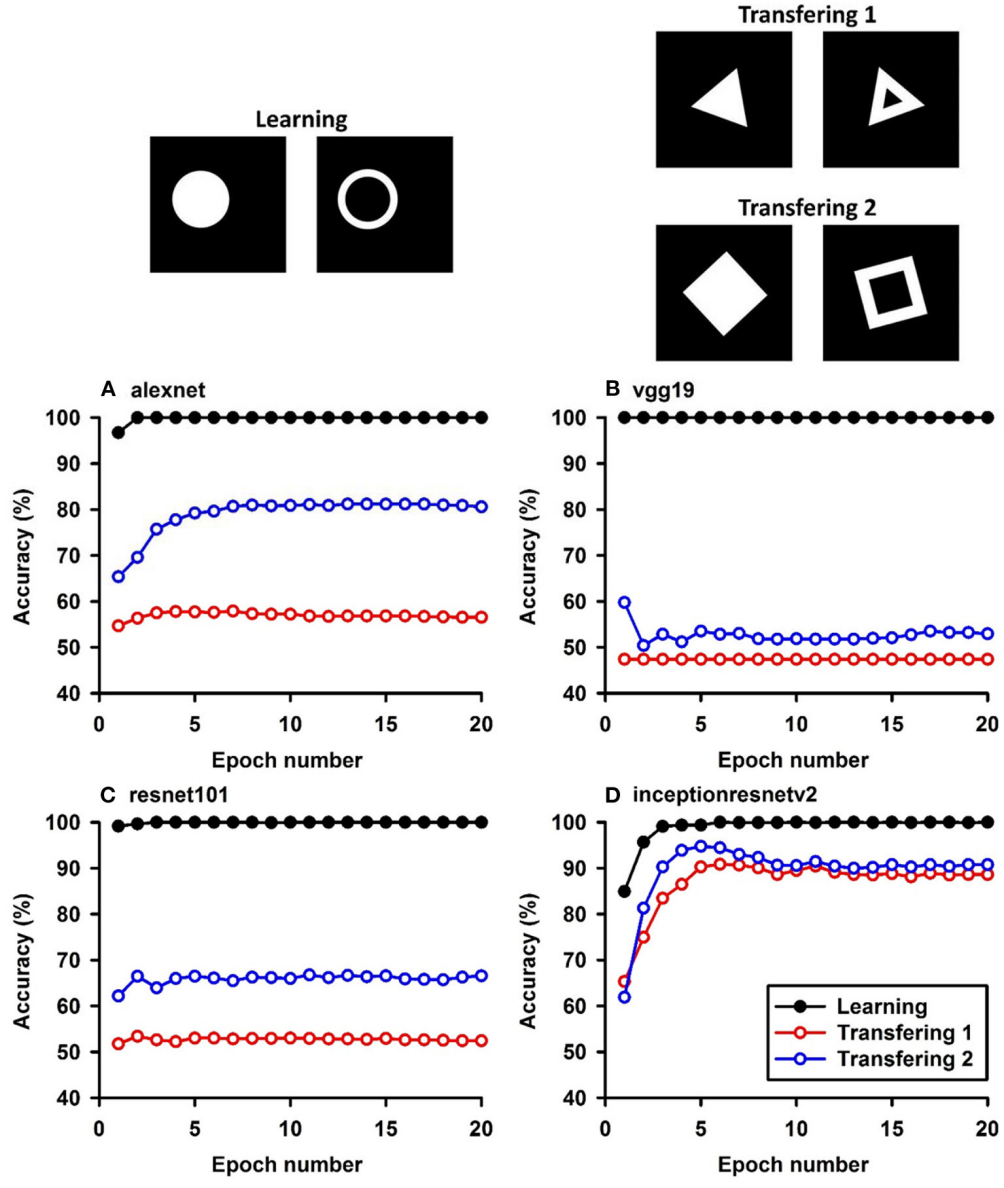
Experiment C. The top panels show the images used in learning and transfer tasks. The lower panel is learning and transfer accuracies as a function of training epochs for the four CNN models.

**Figure 4:**
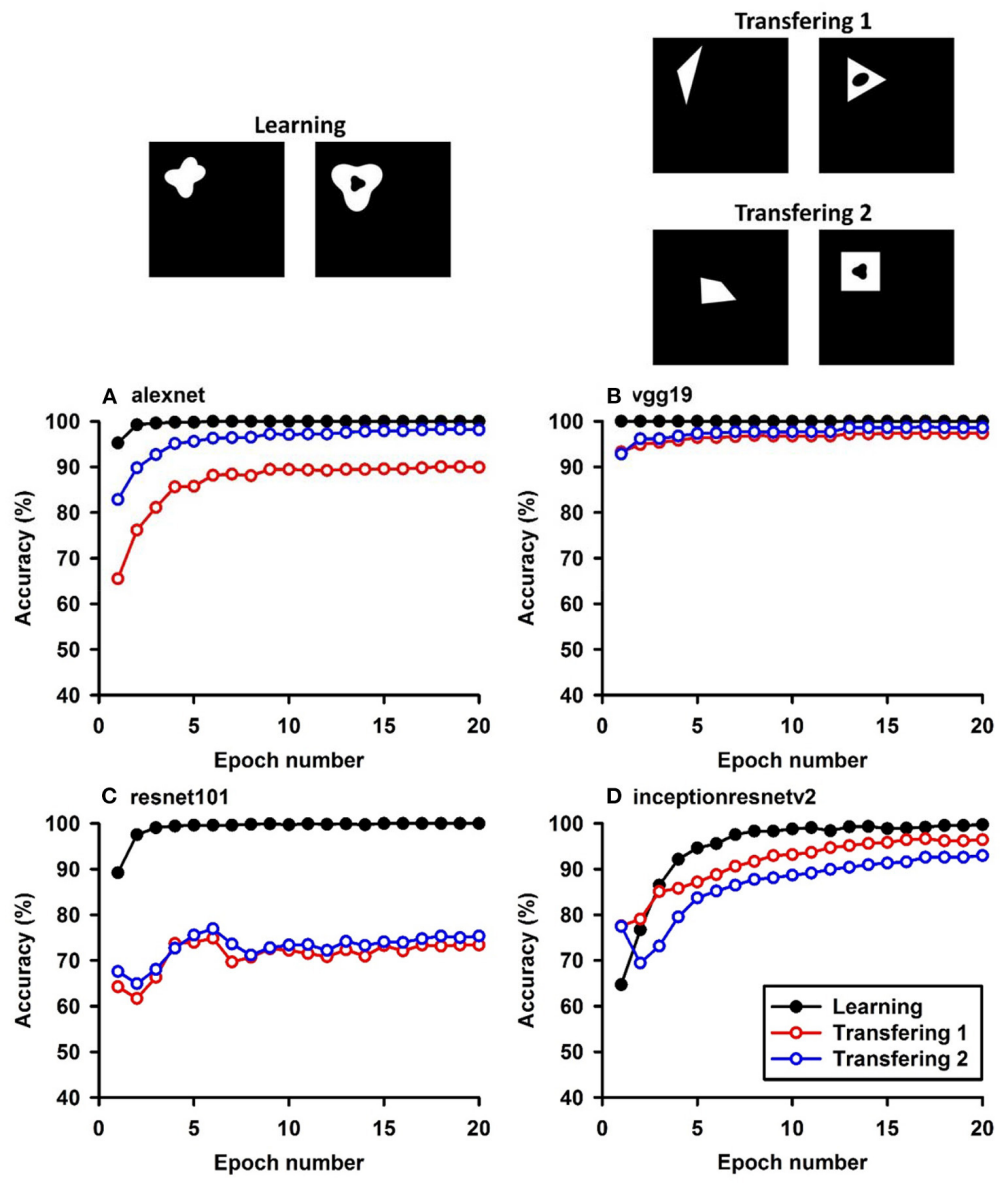
Experiment D. The top panels show the images used in learning and transfer tasks. The lower panel is learning and transfer accuracies as a function of training epochs for the four CNN models.

**Figure 5:**
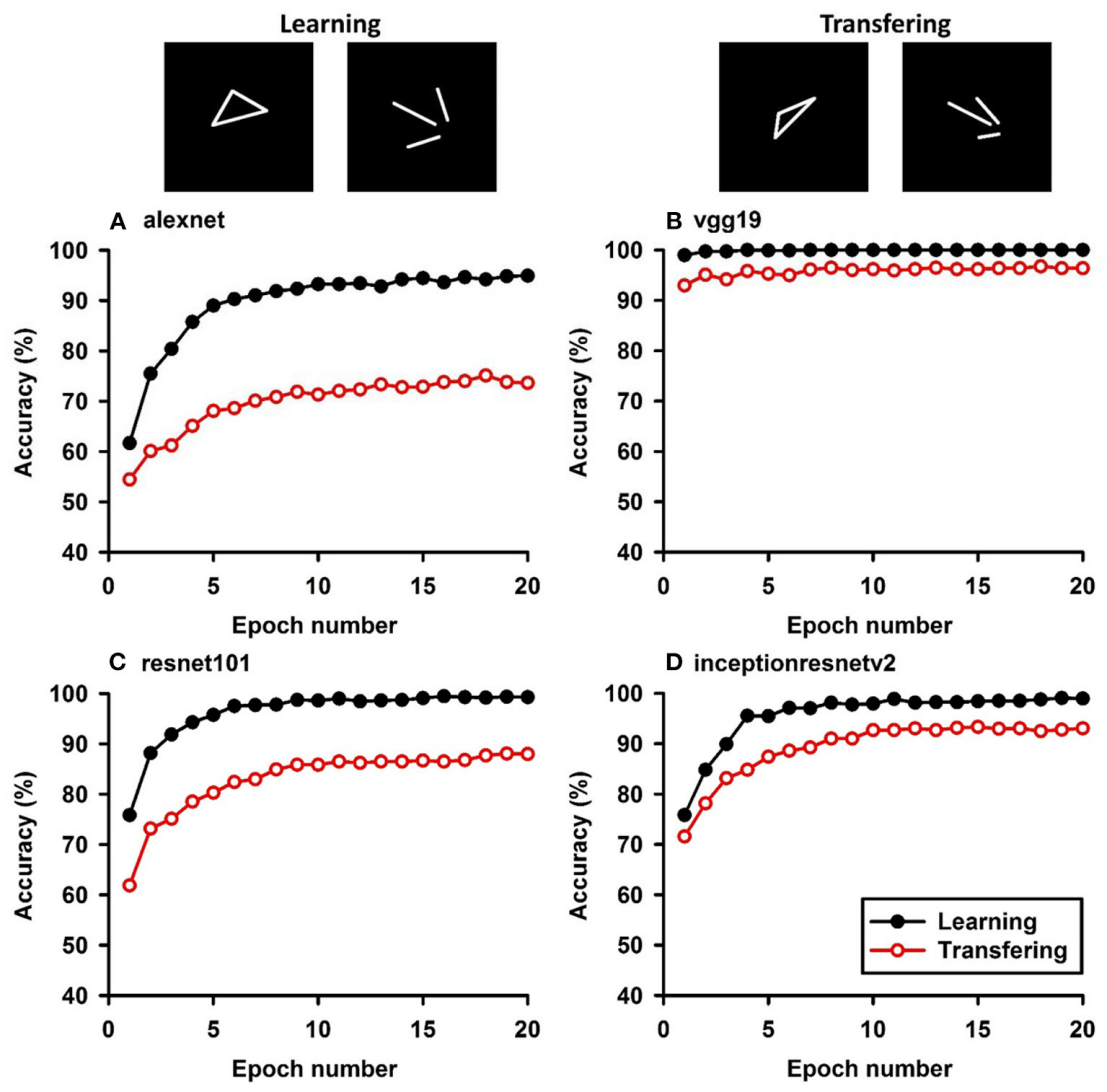
Experiment E. The top panels show the images used in learning and transfer tasks. The lower panel is learning and transfer accuracies as a function of training epochs for the four CNN models.

**Figure 6:**
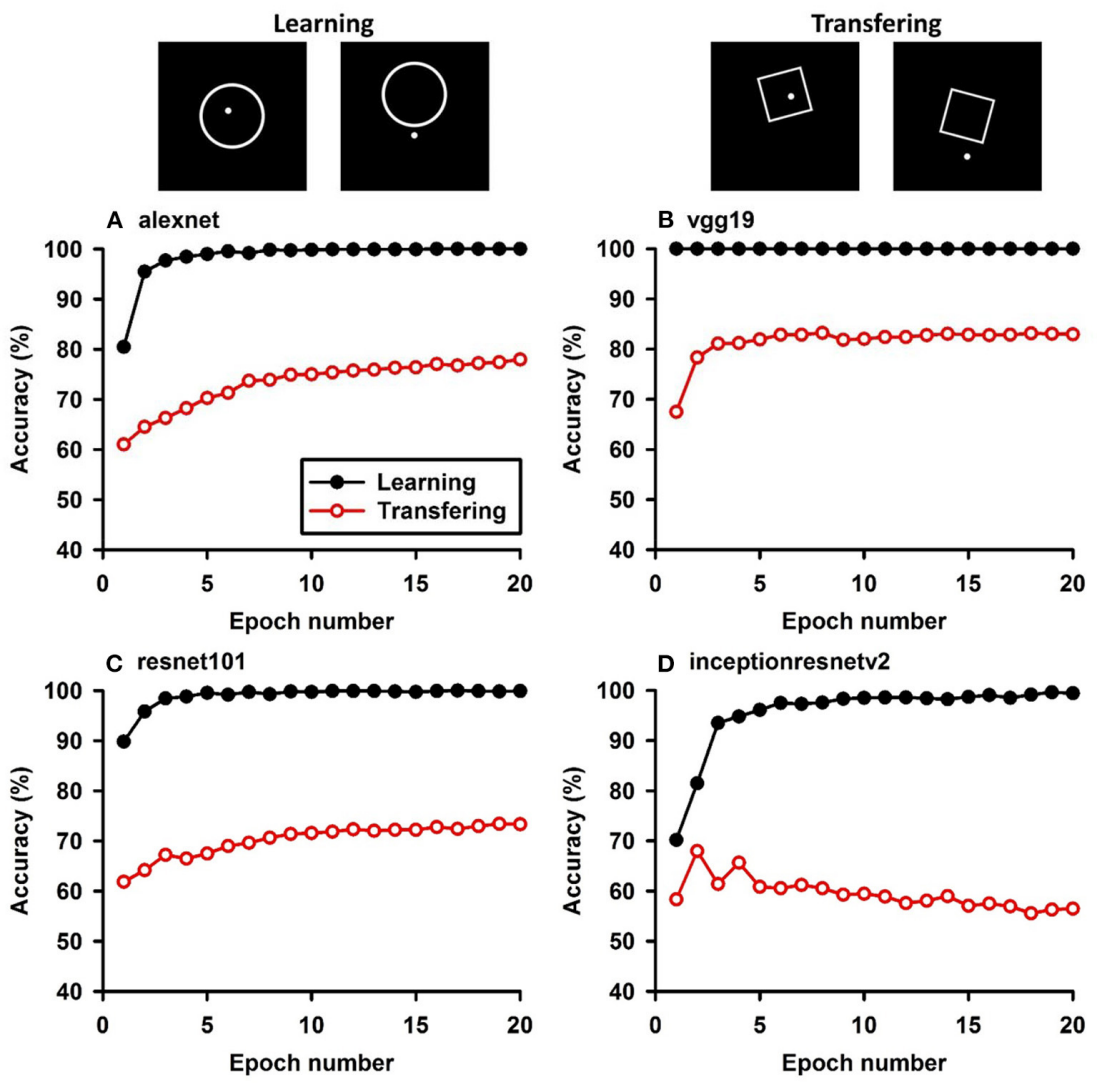
Experiment F. The top panels show the images used in learning and transfer tasks. The lower panel is learning and transfer accuracies as a function of training epochs for the four CNN models.

To quantitatively evaluate transfer of learning (Figures 1–6), we define *transfer index* (TFI) as

(1) $$\begin{array}{r} TFI=HAUC_{Transfer}/HAUC_{Learn}\times 100\%, \end{array}$$

where the Half Area Under Curve (*HAUC*) is calculated using the (*Accuracy* 50) and *Epoch number*. In general, we are interested in classifiers with accuracies higher than 50%. Using (*Accuracy* 50) instead of *Accuracy* is also for normalization purpose. *HAUC*_*Transfer*_ can be negative. The higher the *TFI*, the higher the transfer of learning of a CNN. A perfect transfer, *TFI* = 100% can be achieved when both transfer accuracy and learning accuracy are 100%.

In the following discussion and all tables (Tables 3–8), we use the TFI values to measure the performance of transfer learning. The bold TFI values in each table indicate the best CNN model in that experiment.

**Table 3:** Exp. A. Learning accuracies and transfer index (TFI, percentage) of the four CNNs (Epoch 1 and 20) and the slope and R of the regression.

**Epoch#**\**CNN**	**AlexNet**			**VGG-19**			**ResNet-101**			**Inception-ResNet-v2**		
1 (learning)	69.44			76.94			69.91			63.70		
20 (learning)	75.28			93.43			90.09			91.20		
Transfer Exp.	A.1	A.2	A.3	A.1	A.2	A.3	A.1	A.2	A.3	A.1	A.2	A.3
1 (TFI)	4.78	7.15	−2.37	12.03	3.45	8.57	18.58	13.01	5.58	41.24	45.26	−4.09
20 (TFI)	22.35	4.39	17.96	46.49	22.82	23.67	53.36	24.94	28.41	**62.94**	**42.26**	**20.68**
Slope of regression	0.738	0.029	0.708	0.868	0.548	0.320	0.809	0.366	0.443	0.733	0.462	0.271
R of regression	0.959	0.163	0.943	0.990	0.989	0.921	0.981	0.952	0.974	0.987	0.966	0.912

*The bold values denote the best CNN model corresponding to the highest TFI values in each experiment*.

**Table 4:** Exp. B. Learning accuracies and transfer index (TFI, percentage) of the four CNNs (Epoch 1 and 20) and the slope and R of the regression.

**Epoch#**\**CNN**	**AlexNet**			**VGG-19**			**ResNet-101**			**Inception-ResNet-v2**		
1 (learning)	70.00			73.98			70.00			59.07		
20 (learning)	83.43			100.00			97.13			79.54		
Transfer Exp.	B.1	B.2	B.3	B.1	B.2	B.3	B.1	B.2	B.3	B.1	B.2	B.3
1 (TFI)	19.45	42.60	23.15	4.63	18.14	13.51	8.80	19.45	10.65	50.06	9.15	−40.79
20 (TFI)	20.49	36.82	16.33	**47.40**	**61.30**	**13.88**	41.65	36.16	−5.50	38.25	44.82	6.57
Slope of regression	0.435	0.483	0.048	0.766	0.941	0.175	0.723	0.566	−0.157	0.341	0.581	0.240
R of regression	0.692	0.780	0.223	0.955	0.983	0.876	0.970	0.974	0.822	0.880	0.970	0.873

*The bold values denote the best CNN model corresponding to the highest TFI values in each experiment*.

**Table 5:** Experiment C. Learning accuracies and transfer index (TFI, percentage) of the four CNNs (Epoch 1 and 20) and the slope and R of the regression (N/A mean not applicable).

**Epoch#**\**CNN**	**AlexNet**		**VGG-19**		**ResNet-101**		**Inception-ResNet-v2**	
1 (learning)	96.76		100.00		99.17		84.91	
20 (learning)	100.00		100.00		100.00		100.00	
Transfer Exp.	C.1	C.2	C.1	C.2	C.1	C.2	C.1	C.2
1 (TFI)	10.01	32.93	−5.26	19.50	3.56	24.77	43.83	34.06
20 (TFI)	13.06	61.20	−5.26	5.84	4.88	33.14	**77.20**	**81.48**
Slope of regression	0.729	4.436	N/A	N/A	0.724	3.767	1.690	1.980
R of regression	0.754	0.758	N/A	N/A	0.412	0.707	0.940	0.967

*The bold values denote the best CNN model corresponding to the highest TFI values in each experiment*.

**Table 6:** Experiment D. Learning accuracies and transfer index (TFI, percentage) of the four CNNs (Epoch 1 and 20) and the slope and R of the regression.

**Epoch#**\**CNN**	**AlexNet**		**VGG-19**		**ResNet-101**		**Inception-ResNet-v2**	
1 (learning)	95.27		100.00		89.20		64.68	
20 (learning)	100.00		100.00		100.00		99.72	
Transfer Exp.	D.1	D.2	D.1	D.2	D.1	D.2	D.1	D.2
1 (TFI)	34.26	72.61	86.58	85.64	36.33	44.87	87.67	86.85
20 (TFI)	79.86	96.30	**94.68**	**97.22**	46.76	50.70	93.34	86.36
Slope of regression	5.218	3.188	N/A	N/A	0.909	0.721	0.584	0.610
R of regression	0.907	0.909	N/A	N/A	0.641	0.584	0.911	0.813

*The bold values denote the best CNN model corresponding to the highest TFI values in each experiment*.

**Table 7:** Experiment E. Learning accuracies and transfer index (TFI, percentage) of the four CNNs (Epoch 1 and 20) and the slope and R of the regression.

**Epoch#**\**CNN**	**AlexNet**	**VGG-19**	**ResNet-101**	**Inception-ResNet-v2**
1 (learning)	61.67	98.98	75.83	75.83
20 (learning)	94.91	100.00	99.26	98.98
1 (TFI)	38.05	87.71	45.88	83.51
20 (TFI)	52.57	**92.78**	77.06	87.91
Slope of regression	0.639	3.514	1.134	0.967
R of regression	0.967	0.897	0.976	0.967

*The bold values denote the best CNN model corresponding to the highest TFI values in each experiment*.

**Table 8:** Experiment F. Learning accuracies and transfer index (TFI, percentage) of the four CNNs (Epoch 1 and 20) and the slope and R of the regression.

**Epoch#**\**CNN**	**AlexNet**	**VGG-19**	**ResNet-101**	**Inception-ResNet-v2**
1 (learning)	80.46	100.00	89.81	70.19
20 (learning)	100.00	100.00	99.91	99.44
1 (TFI)	36.18	35.00	29.77	41.26
20 (TFI)	55.92	**65.92**	46.74	13.11
Slope of regression	0.838	N/A	1.138	−0.167
R of regression	0.764	N/A	0.824	0.393

*The bold values denote the best CNN model corresponding to the highest TFI values in each experiment*.

### Classification With Local Features: Different Shapes

In Experiment A, the CNNs were trained to discriminate squares vs. trapezoids. If the classification was based on angles of neighboring sides or parallelism of opposing sides, we expect the models to classify rectangles vs. trapezoids as squares vs. trapezoids in Experiment A.1 (see the red curves labeled “Transferring 1” in Figure 1). In general, we expect that a trained model recognizes Column 1 images in the transfer dataset as Colum 1 images in the training dataset (Table 2) if the shared geometric invariants were used for classification. Note that the parallelograms appear in both columns, which partially explains that the accuracies of Transfer 2 and Transfer 3 are lower than that of Transfer 1 (Figure 1 and Table 3). The Inception-ResNet-v2 model performed the best and achieved 62.94, 42.26, and 20.68% of transfer index on the three transfer tests (Table 3).

In Experiment B, squares vs. parallelograms were used to train the CNNs. Trapezoids were listed in both columns, which caused lower accuracies of Transfer 1 and Transfer 3 (shown in red and green curves in Figure 2). The VGG-19 model was the best and reached 47.4, 61.3, and 13.88% of transfer index (Table 4). The low transfer accuracies indicate that similarity extraction were not complete. Transfer of learning was the worst when classifying two unseen shapes (Transfer 3 in Experiment A and Experiment B, green curves in Figures 1, 2). Note that the transfer curves were in “parallel” with the learning curves, indicating the CNNs did extract similarities between the training dataset and the transfer datasets. Linear regressions were performed to quantify the relationship between learning accuracy and transfer accuracy (Supplemental Materials). Slope and R of the regressions were used to assess the correlation between transfer accuracy and learning accuracy.

### Classification With Global Features: No-Hole vs. One-Hole

The CNNs were trained to discriminate disks (no-hole) vs. rings (one-hole), and transfer of learning was tested on triangles vs. triangle-rings in Experiment C.1 and squares vs. square-rings in Experiment C.2, respectively. A perfect transfer would be expected if the presence of a hole was used to perform the classification. The Inception-ResNet-v2 performed the best (Figure 3) and achieved 77.2 and 81.48% of transfer index for the two transfer tasks, respectively (Table 5).

Similar tests with irregular shapes were conducted in Experiment D. The VGG-19 model had high transfer index of 94.68 and 97.22% for the two transfer tasks, respectively (Figure 4 and Table 6). The high transfer performance were impressive when considering the fact of that the model had never been exposed to shapes in the transfer datasets, indicating that presence of a hole (a topological invariant) was likely extracted and used for classification.

Note that the transfer index values of irregular shapes are higher than that of regular shapes. More experiments are needed to identify the underlying mechanisms.

### Classification With Global Features: Connectivity

In Experiment E, the four CNNs were trained with isosceles-triangles (connected) vs. its three sides separated (not connected), and transfer of learning were tested on irregular-triangles vs. its three sides separated. If connectivity (a topological invariant) was extracted during the learning, we would expect high transfer accuracies in this task. Among the four models, VGG-19 exhibited the highest transfer index of 92.78% (Figure 5 and Table 7).

### Classification With Global Features: Inside/Outside Relationship

In Experiment F, the CNNs were trained to discriminate dot-inside-circle vs. dot-outside-circle, and transfer of learning was tested on dot-inside-square vs. dot-outside-square (Figure 6). While the VGG-19 achieved a moderate transfer index of 65.92% (Table 8), the other three models, including the two advanced models, exhibited lower transfer index of 46.74 and 13.11%, respectively, indicating that inside/outside relationship was not extracted for classification during the learning phase.

## Summary and Discussion

In this study, we trained four CNNs to perform shape classification tasks based on local or global features and further examined how learning of classifying shapes in the training datasets was transferred to classifying shapes in the transfer datasets, which share local or global features with the training datasets. Experiments were designed to test two hypotheses on transfer of CNN learning. First, we wanted to test whether learning tasks based on local features have a higher transfer accuracy than that based on global features. This hypothesis was motivated by the local-to-global hierarchical organization of the CNN architecture, where local features are fully extracted and represented by the early layers. Second, we wanted to test whether the advanced CNNs have higher transfer accuracy for learning tasks based on global features than the early CNNs. This hypothesis was motivated by the fact that the advanced CNNs employ more layers and recurrent connections, which had advantages of extracting global features by integrating inputs from a large region. Although this is a pilot study using the ShapeNet approach, our results provide clear evidence that does not support the two hypotheses.

As expected, the CNNs performed well in the learning tasks, regardless classifying shapes using local features (Experiments A and B) or global features (Experiments C–F). After 20 epochs of training, they classified the shapes in the training datasets at high accuracies (>95%), indicating feasibility of employing pre-trained CNNs to learn new tasks on a small dataset. However, their performance in the transfer experiments varied from task to task, and from model to model. Regarding the first hypothesis, we found that transfer accuracies for local features (Experiments A and B) were lower than those with global features (Experiments C–F). In the example of Resnet101, after it was trained to discriminate squares from trapezoids, they were tested to discriminate rectangles from trapezoids. The squares share many local features with squares, such as four angles of 90 degrees, two pairs of sides parallel to each other, etc. If the model learned to discriminate the pair of shapes based on these shared features, we should expect a perfect transfer, TFI = 100%. Contrary to this prediction, we found that Resnet101 only had 53.36% transfer to rectangles and 24.94% transfer to parallelograms. On the other hand, after Resnet-101 was trained to discriminate regular triangle from their separated sides, they were tested to discriminate irregular triangles from their separated sides. If Restnet101 learned to discriminate connected shape from disconnected shapes (i.e., connectivity, a topological invariant), we would expect to observe a high transfer accuracy. Indeed, it showed a transfer index of 77.06%, much higher than the transfer accuracy based on local features. This finding is counterintuitive, suggesting a lack of understanding of the mechanisms underlying CNN image classification. However, the ShapeNet analysis provides a quantitative approach to gain insight into this difficult problem. Future studies will systematically manipulate the differences between the learning datasets and the transfer datasets to tease out the features used in the learning tasks.

**Figure 7:**
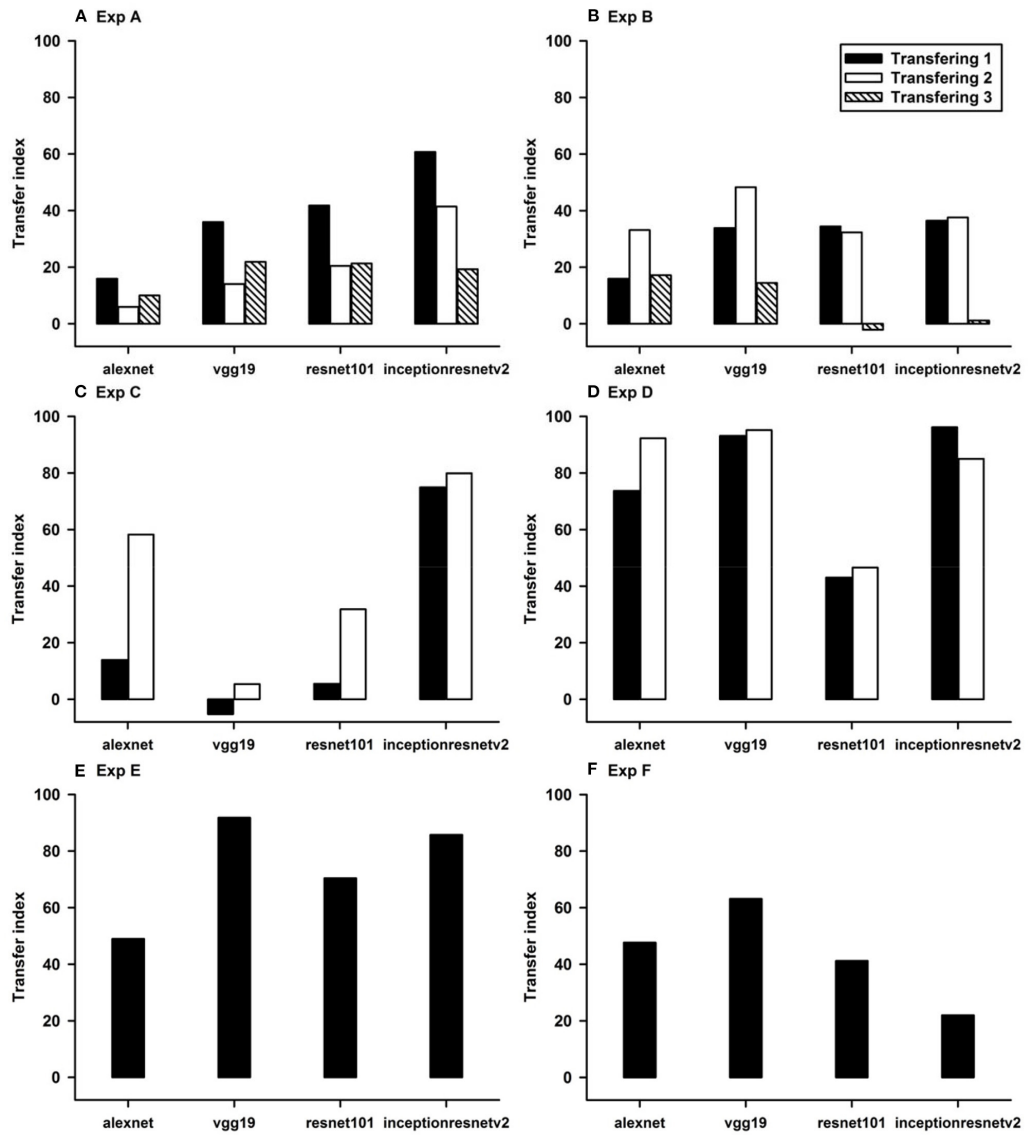
Evaluation and comparison of transfer learning of the four CNN models using transfer index.

Regarding the second hypothesis, we found that the more advanced CNNs do not have higher transfer accuracies based on global features. For example, Inception-ResNet-v2 has 192 layers and VGG19 has 19 layers. However, VGG19 exhibited higher transfer accuracies on learning based on global features, such as connectivity (Table 7, 92.78 vs. 87.91%) and inside-outside relationship (Table 8, 65.92 vs. 13.11%). Among the three global (topological) invariants, we found that inside/outside relationship had the lowest transfer performance in the CNNs (Figure 7). In the training datasets of dot-inside-circle/dot-outside-circle, circle size, circle position and dot position with respect to the circle varied from image to image. The high learning accuracies (>99%) indicate that the CNNs successfully extracted the common features of the learning datasets. However, after only replacing circle by square, the models performed poorly in classifying dot-inside-square and dot-outside-square. The most advanced CNN model only had a transfer index of 13.11%. This counterintuitive result suggests that the CNNs achieved shape classification by adopting different strategies than extracting inside/outside relationship. Note that different from the other tasks, where the transfer curves are in parallel with the learning curves, indicating extracting shared properties between the training datasets and the transfer datasets (Supplementary Figures 1, 2), the transfer curve for the Inception-ResNet-v2 did not increase in parallel with the learning curve. In fact, the correlation coefficient between the transfer accuracy and learning accuracy was −0.17. Among the three tested global features, inside-outside relationship seems to be a limitation of the CNNs, which is not overcome by increasing depth and recurrent connections. This task may be an effective benchmark for developing new CNNs that can extract global features under various conditions.

In summary, this pilot study presented a proof of concept of the “ShapeNet” approach that can be used to elucidate the mechanisms underlying CNN image classification. Rejecting the two intuitive hypotheses indicate clear knowledge gaps in our understanding of CNN image processing. Since the same stimuli and tasks can be used to study visual information processing in humans and monkeys, the “ShapeNet” approach may be an effective platform to compare CNN vision and biology vision. In fact, in addition to the well-known local-to-global approach, there are accumulating evidence for an alternative global-to-local approach, such as object-superiority (Weisstein and Harris, 1974), early detection of topological properties (Chen, 1982, 1990), and rapid processing of global features in non-human primates (Huang et al., 2017). While the exact underlying mechanisms and differences between CNN models and primate visual systems are unknown, the results suggested that the primate visual systems process local and global features in different ways than the CNNs. By recognizing the differences, future studies will be focused on extending the analysis to other local/global geometrical invariants to understand the CNNs and the biological visual functions. In particular, we will test how humans and monkeys transfer their learning based on inside/outside relationships (topological invariant). We believe comparison between the CNN vision and biological vision using the “ShapeNet” approach will provide insight into a better understanding of visual information processing in both systems.

## Data Availability Statement

The raw data supporting the conclusions of this article will be made available by the authors, without undue reservation is available at http://r2image.com/transfer_learning/datasets.zip.

## Author Contributions

YZ analyzed all images with four CNN models and drafted the sections regarding CNN method and models. JH created all geometric images and figures. He also analyzed CNN results. TC and YO assisted in running all experiments. WZ designed all experiments and drafted the sections regarding hypotheses and result discussion. All authors contributed to the article and approved the submitted version.

## Conflict of Interest

The authors declare that the research was conducted in the absence of any commercial or financial relationships that could be construed as a potential conflict of interest.

## Footnotes

^1^Very Deep Convolutional Networks for Large-Scale Visual Recognition, http://www.robots.ox.ac.uk/\simvgg/research/very_deep/.
