# Supplementary material for: Transfer of Learning in the Convolutional Neural Networks on Classifying Geometric Shapes Based on Local or Global Invariants
Source: Front Comput Neurosci. 2021 Feb 19;15:637144. doi: 10.3389/fncom.2021.637144 (PMC7935523; doi:10.3389/fncom.2021.637144)
Supplement: Supplementary file 1 [file Data_Sheet_1.PDF]

Supplementary Material

1 Evaluation of Transferring Classification

To evaluate the correlation between learning and transfer, the transfer-learning accuracy was analyzed with linear regression. The results of linear equations are presented in Supplementary Figures S1-6.

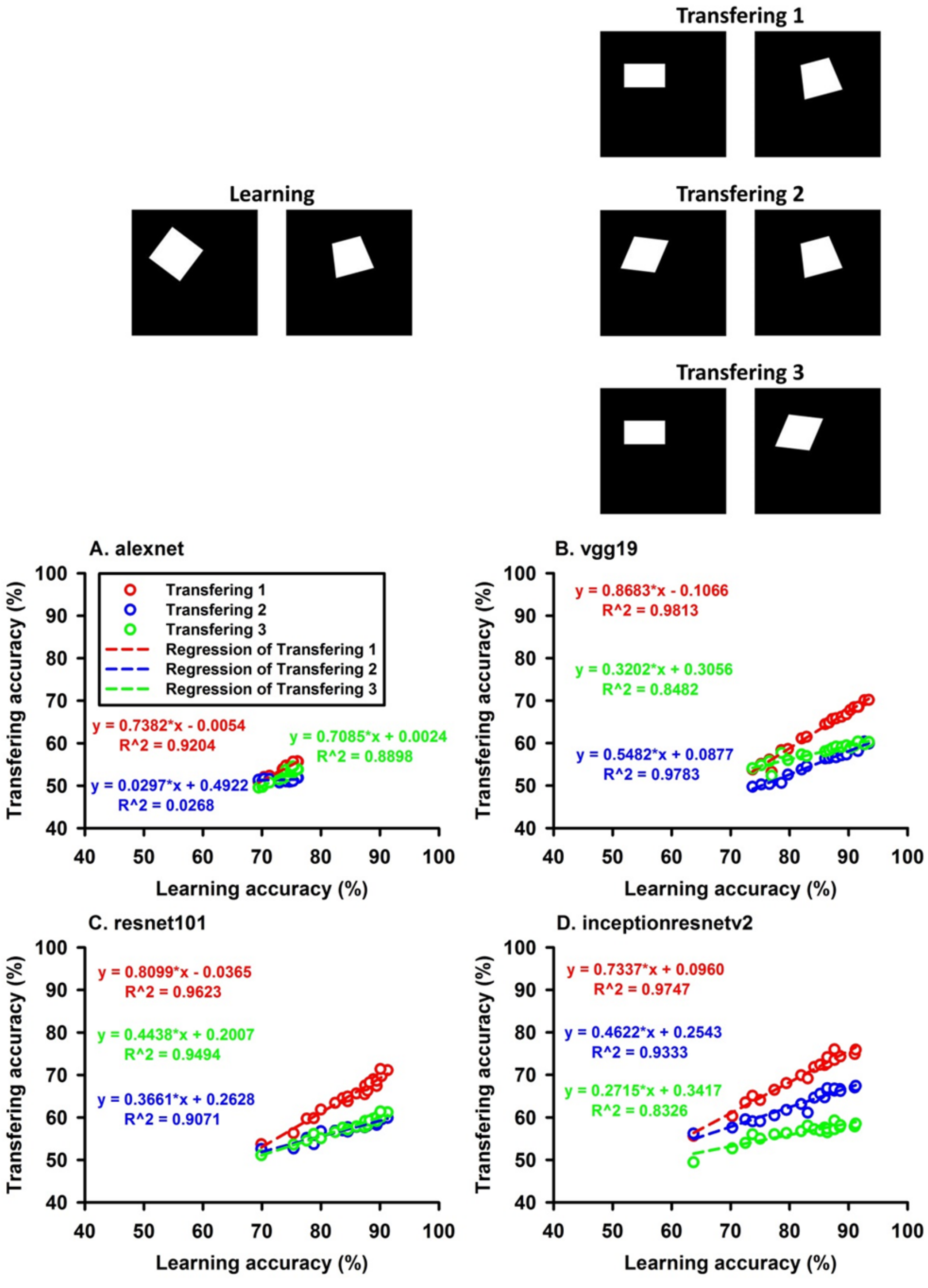

Figure S1. Correlations between transfer accuracy and learning accuracy (circles) in Exp. A.

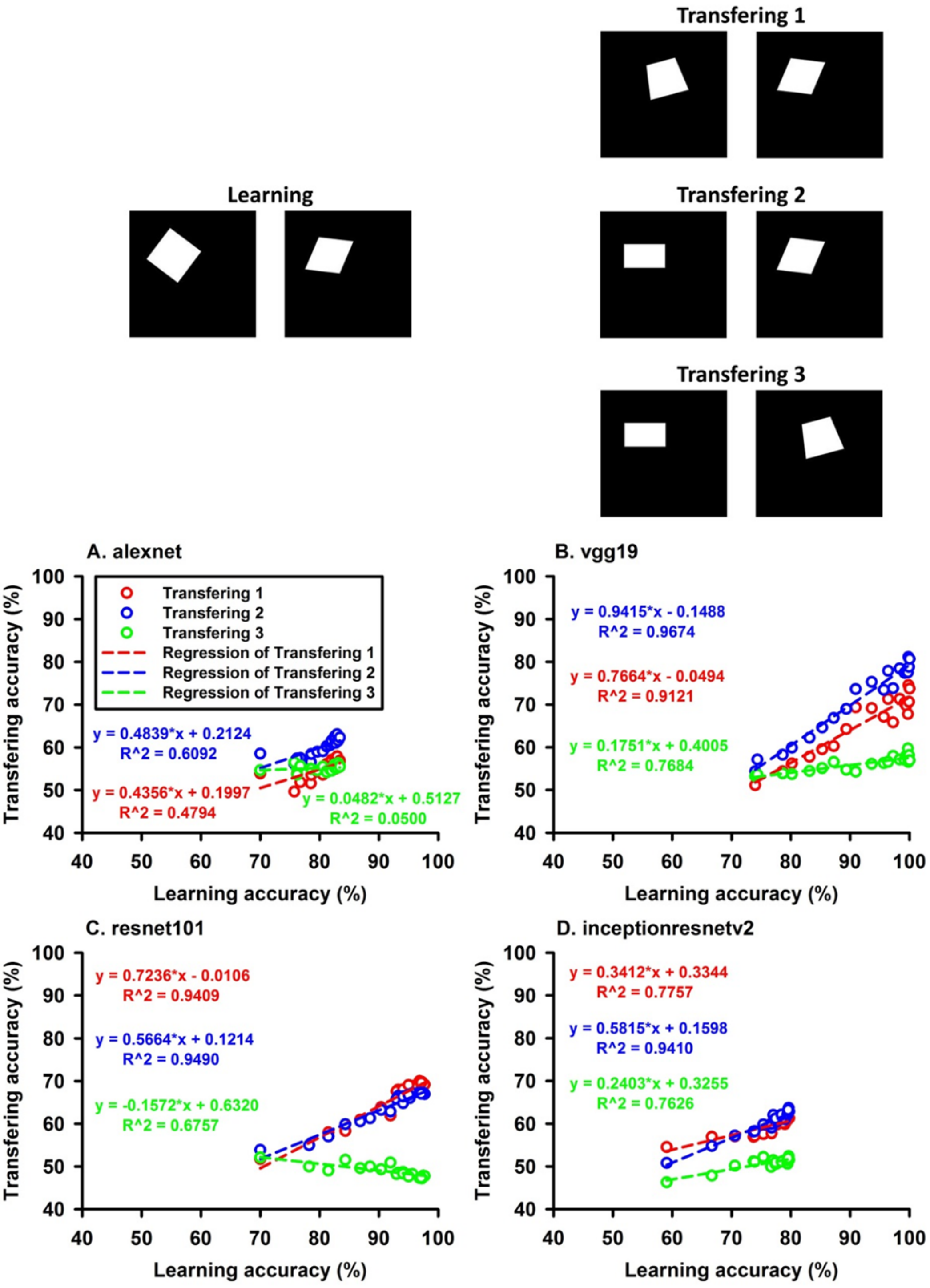

Figure S2. Correlations between transfer accuracy and learning accuracy (circles) in Exp. B.

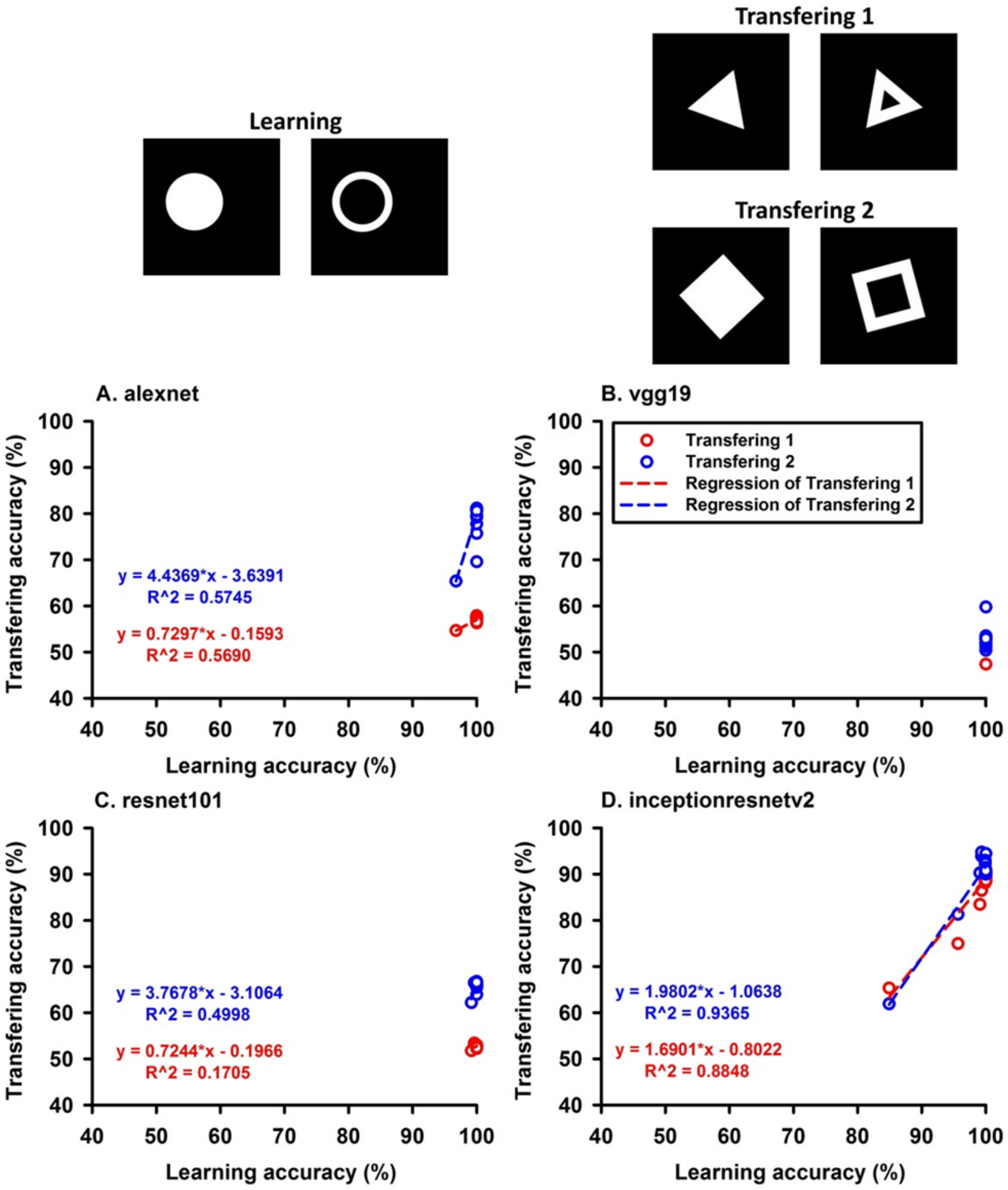

Figure S3. Correlations between transfer accuracy and learning accuracy (circles) in Exp. C. No linear regression was done in subplot B (vgg19) due to lack of variation in the learning accuracy.

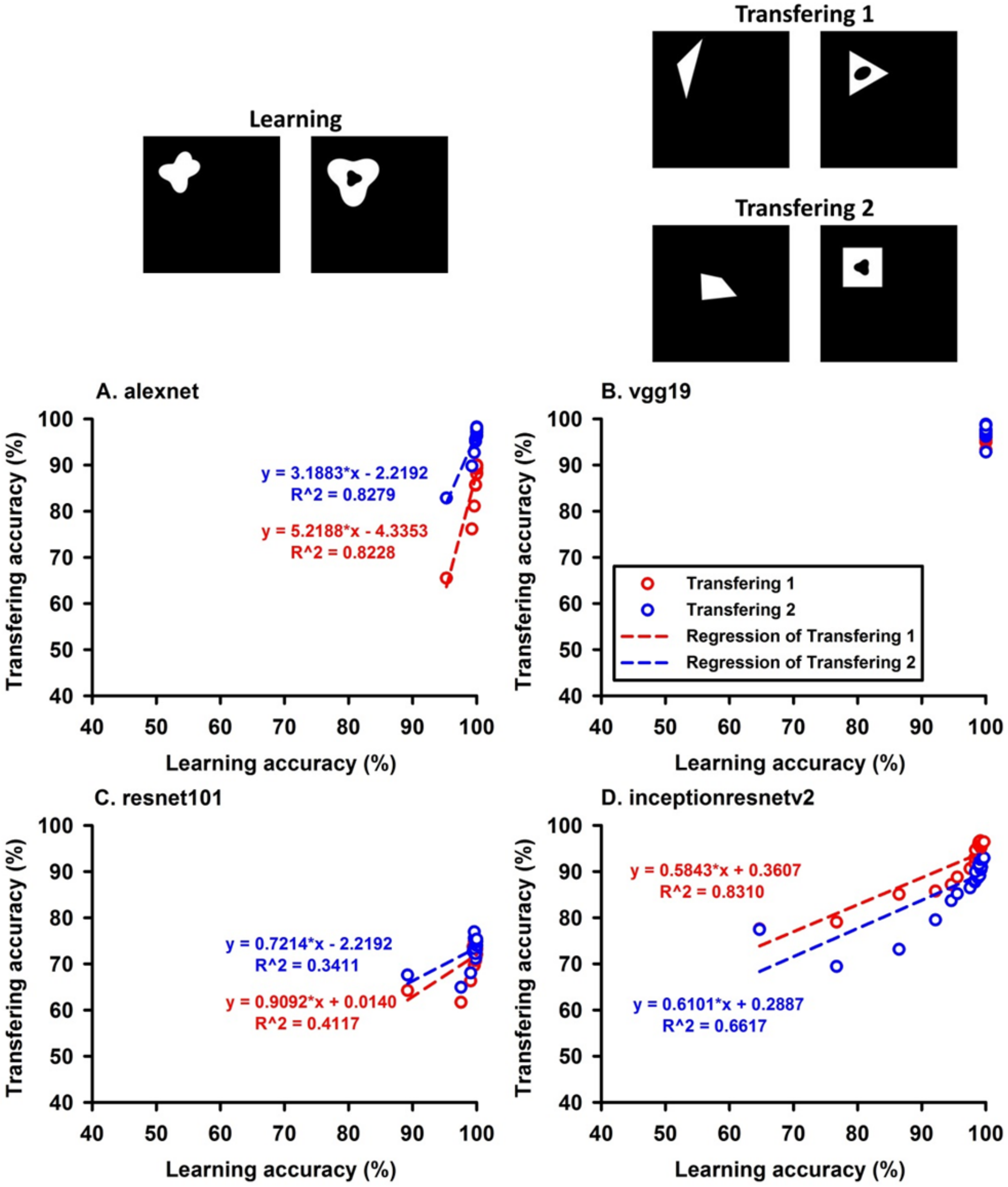

Figure S4. Correlations between transfer accuracy and learning accuracy (circles) in Exp. D. No linear regression was done in subplot B (vgg19) due to lack of variation in learning accuracy.

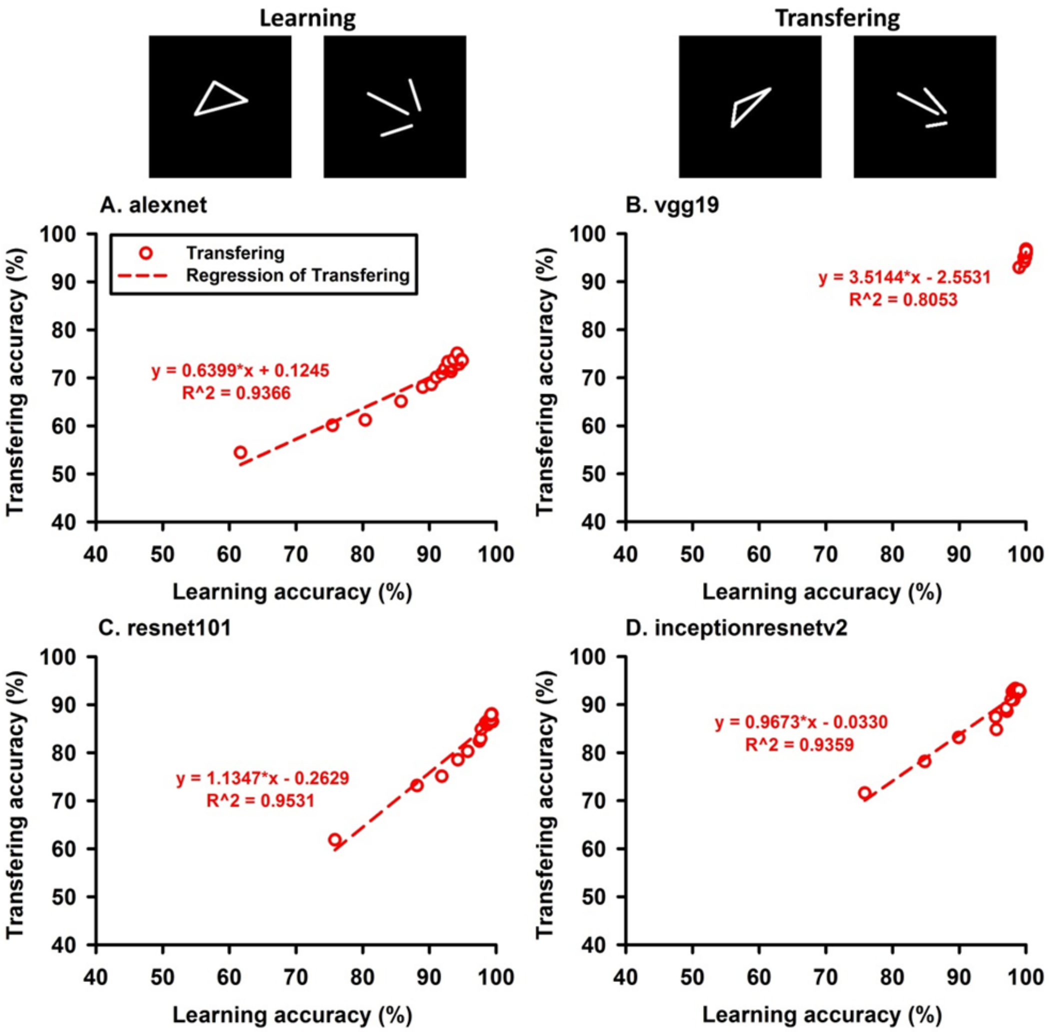

Figure S5. Correlations between transfer accuracy and learning accuracy (circles) in Exp. E.

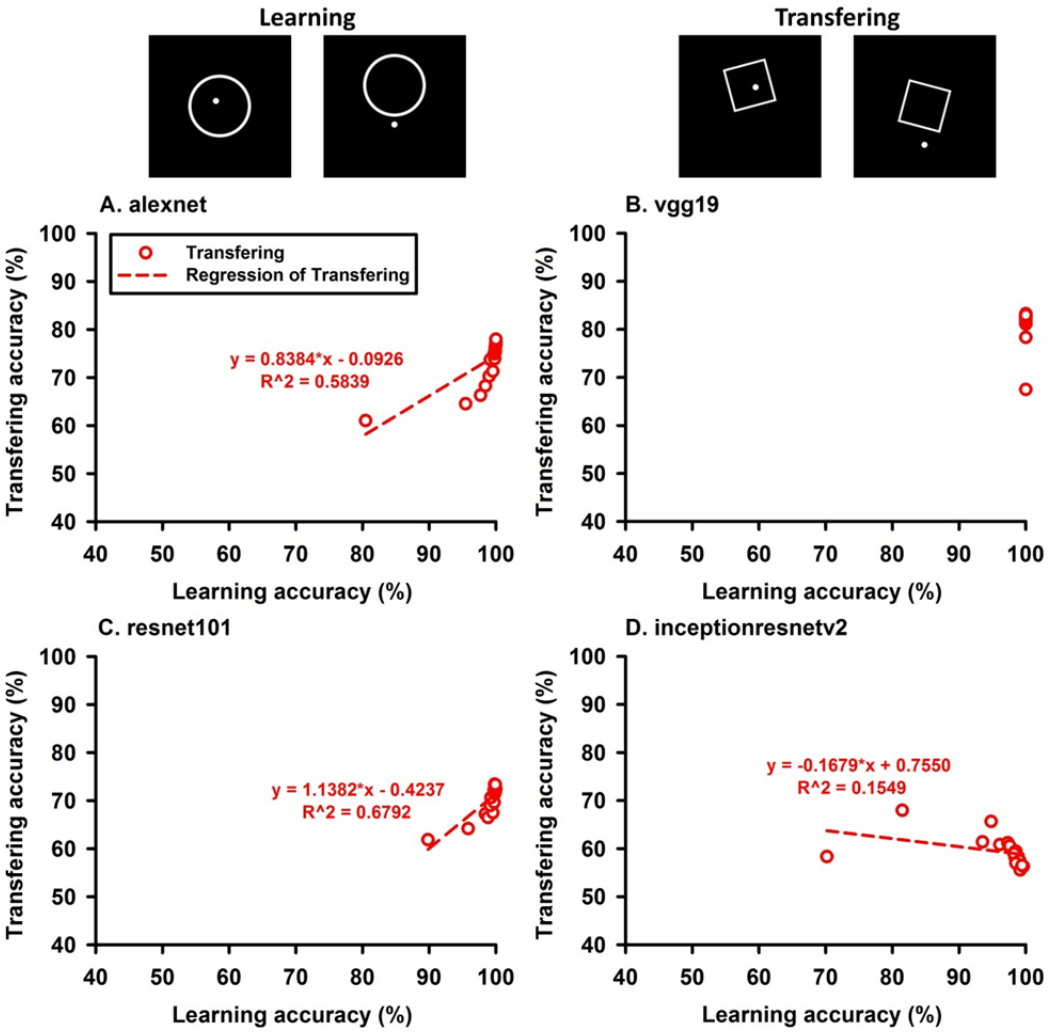

Figure S6. Correlations between transfer accuracy and learning accuracy (circles) in Exp. F. No linear regression was done in subplot B (vgg19) due to lack of variance in learning accuracy.
